# Declining genetic diversity of European honeybees along the twentieth century

**DOI:** 10.1038/s41598-020-67370-2

**Published:** 2020-06-29

**Authors:** Gonçalo Espregueira Themudo, Alba Rey-Iglesia, Lucía Robles Tascón, Annette Bruun Jensen, Rute R. da Fonseca, Paula F. Campos

**Affiliations:** 1 CIIMAR, Interdisciplinary Centre of Marine and Environmental Research, University of Porto, Avenida General Norton de Matos, S/N, 4450-208 Matosinhos, Portugal; 2 Section of Forensic Genetics, Department of Forensic Medicine, Faculty of Health Sciences, University of Copenhagen, Frederik V’s vej 11, 2100 Copenhagen, Denmark; 3 Institute of Biological Psychiatry, Mental Health Centre Sankt Hans, Capital Region of Denmark, 4000 Roskilde, Denmark; 4 Centre for GeoGenetics, Natural History Museum of Denmark, University of Copenhagen, Copenhagen, Denmark; 5 The Bioinformatics Centre, Department of Biology, University of Copenhagen, Copenhagen, Denmark; 6 Department of Plant and Environmental Sciences, University of Copenhagen, Thorvaldsensvej 40, 1871 Frederiksberg C, Denmark; 7 Center for Macroecology, Evolution and Climate, Natural History Museum of Denmark, University of Copenhagen, Copenhagen, Denmark

**Keywords:** Genetic variation, Genomics

## Abstract

The European honeybee (*Apis mellifera*) is a key pollinator and has in the last decades suffered significant population decline. A combination of factors, including decrease in genetic diversity and introduction of *Varroa* mites, have been suggested to be responsible for these losses, but no definitive cause has yet been appointed. In Europe not only have wild colonies been severely affected, but managed hives have had a massive decline in numbers. To test the hypothesis that honeybees’ genetic diversity has decreased in the recent past, we used reduced representation genome sequencing of 40 historical honeybee specimens collected in Natural History collections across Europe and compared them to genomic data from 40 individuals from extant populations (collected post 2006). Our results are consistent with the existence of five evolutionary lineages as previously described, and show a decrease in genetic diversity between historical and extant individuals of the same lineage, as well as high levels of admixture in historical specimens. Our data confirm that a loss of genetic diversity has occurred during the last century, potentially increasing honeybees’ vulnerability to contemporary ecological and anthropogenic stressors.

## Introduction

Honeybees are one of the most important pollinator species and the most widely used insect, managed for its pollination services and production of honey. The number of managed honeybee colonies in Europe has generally decreased since the 1960′s, at least in Central Europe^1^. The number of wild or feral honey bees is less known, with many believing or assuming that they no longer exist. Recent work however suggests that feral honey bees still colonize beech forests in Germany, and probably much of Central Europe^2^.

Even though bees have been used to produce honey and for pollination purposes for over 7,000 years, since at least Ancient Egypt civilizations^3–5^, it was only when beekeeping techniques were perfected in the seventeenth and eighteenth centuries that it became possible to maintain large bee colonies giving rise to modern apiculture^6^. More recent practices, such as the commercial mass rearing of queens, artificial selection of behaviours favouring honey production, and the presence of thousands of bees in limited spaces, may have altered the natural processes and affected the genetic diversity of domestic and wild (or feral) hives, increasing their susceptibility and the transmission rate of diseases between bees^7^. There is an ongoing debate about whether European honeybees are domesticated (in the sense that selective breeding over generations has led to artificial selection) or not^8–10^. Traits favourable to beekeepers, such as docility, lack of propensity to swarming, honey yield, and others may be selected for, but as it is difficult to have controlled mating, this is usually done through the import of stock from other areas, where these traits are more frequent. This has consequences for wild populations, as due to the wide freedom honeybees have even when in artificial hives^9^, factors influencing one of them will have a similar effect on the other.

**Figure 1:**
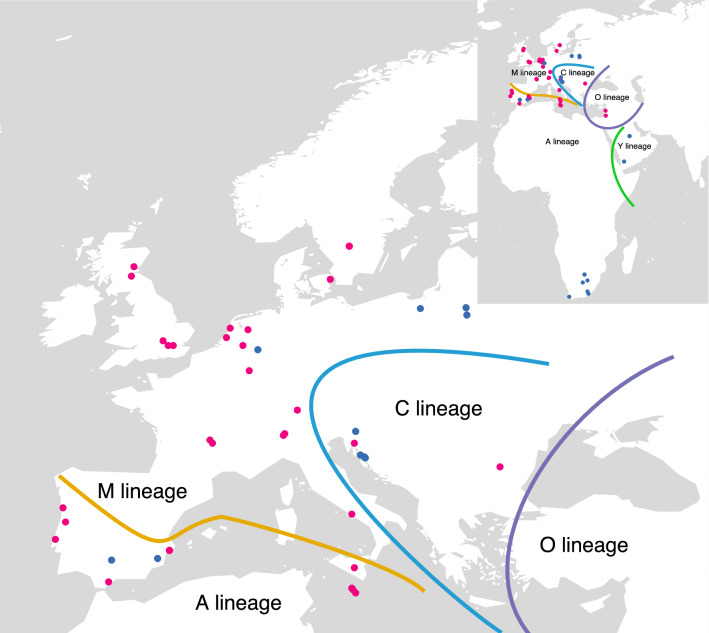
Map showing approximate limits of the A, C, M, O, and Y evolutionary lineages of *Apis mellifera*, and original sampling locations of historic (red) and contemporary (blue) specimens used in this study.

There are at least 28 subspecies of *Apis mellifera*^11,12^ described based on geography and morphological variation; morphometric and genetic studies have consistently showed that there are only four or five major evolutionary lineages, with one or two of them only occurring in Africa. Honeybees were first aggregated into four major lineages (A, C, M, and O) based on morphometry and biogeography^13^. Lineage A is present in Africa and in the Iberian Peninsula, O in the Middle East, M in Northern and Western Europe, and C in South Eastern Europe. The existence of a fifth lineage from north-eastern Africa named Y was proposed and supported using mitochondrial DNA^14^. The approximate distribution of the lineages in Africa, Europe, and Western Asia is shown in Fig. 1. The main hypothesis for the origin of *Apis mellifera* is of an Asian origin, as all other *Apis* species are endemic to Asia. However, genetic diversity is higher in Africa, which has led some authors to speculate on an African origin for the species^14^.

Early genomic work on honeybees focused on molecular determinants in behaviour^15,16^. After the release of the honeybee genome^17^, several specific pathways involved in immunity^18^, and olfaction, and gustation^19^ have been identified.

Considering the important role of honeybees for the pollination of monocultures, that are essential for human food security and variety, and other ecological services performed by honeybees, there is dire need for a better understanding of changes in genetic variability of (semi)natural populations and how these may be related to domestication and artificial selection.

Museum collections are ideal for this purpose, as they provide a temporal series of honeybees from different areas of their natural range, from which trends can be derived, and they are invaluable sources of chitinous material from which DNA can be retrieved without conferring any external damage to the specimen^20–22^. Museum specimens have been widely used to provide insight into past genetic diversity, domestication, taxonomic placement, and migration of several species^23,24^ . However, specimens were not always collected considering the preservation of genetic material, and DNA degrades as a function of temperature and time. For this reason, it is necessary to use ancient DNA (aDNA) techniques to retrieve usable DNA from these specimens. We therefore use the term ancient DNA not as a reference to the age of our specimens, but to the likely state of degradation of the molecules. The low percentage of endogenous DNA (that belonging to the organism, and not contaminating fungi or bacteria) in museum samples still poses limitations to aDNA shotgun sequencing experiments, as most sequences yielded would not be from the specimen. DNA capture-enrichment methods, in contrast, allow targeted sequencing by selective enrichment of sequences of interest prior to sequencing, hence increasing the depth of sequencing over target regions and lowering costs per target^25^.

As honeybees are thought of being at least partially domesticated, they were not usually collected with purpose by naturalists, but rather as incidental bycatches when collecting other insect species thought to have greater natural history value. Curation and annotations were therefore sparse and incomplete. Despite this, several historical collections exist. We took advantage of this, and generated genomic data on historical European honeybee specimens, allowing us to explore the genetic diversity of the species over the last 150 years. In this study, we assess the genetic diversity of honeybees across Europe from different time periods using historical museum collections and ancient DNA (aDNA) techniques.

## Material and methods

### Data collection

Pin-dried specimens of *Apis mellifera* covering most of the natural range of honeybees in Europe were obtained from museum collections. As collections were not digitized at the time of sampling, all information from labels were recorded manually, including when available: date of sampling, location name, geographical coordinates, sex, and name of collector. None of the collections had unique identifiers (voucher numbers) for these specimens, so internal identifiers were used consisting of two letter country code, four-digit year of sampling, and, if more than one specimen matched the country/year combination, one letter (a through e). This is the only information available for these specimens, and we therefore do not know if their origin is from feral, wild or managed colonies. As bees in managed hives can freely move around their area, gene flow between managed and feral hives is most likely unrestricted, so we believe this does not affect the interpretation of our results. Subspecies/lineage information was not known a priori when selecting specimens, so sampling attempted to cover a wide geographic and temporal span.

The time span of the specimens is from 1850 to 2002 (See Fig. 1 and Supplementary Table S1). DNA extraction, Illumina library preparations, and PCR setups were performed in a dedicated ancient DNA laboratory. Total genomic DNA was extracted using the non-destructive method described in Gilbert et al.^20^ and detailed in Campos and Gilbert^21^. Probes for in-solution, hybridization capture enrichment kits (MYcroarray) were designed for randomly selected gene locations present in gene set AmelOGSV3.2^26^, but also for immune^27^, sensory^19^, and behavioural^15^ biochemical pathways (SI Appendix and Supplementary Table S2). Libraries were pooled with other indexed DNA libraries and sequenced on Illumina HiSeq platforms at the Danish National High-Throughput DNA Sequencing Centre. Whole genome sequences of 40 honeybees collected after 2006 from lineages A, C, M, and Y and one *Apis cerana* were retrieved from NCBI’s short read archive^28^.

### Analysis

Sequencing data were analysed using a set of custom scripts and software (SI Appendix). Adapter sequences were trimmed and filtered for N’s and reads shorter than 30 bp were removed using AdapterRemoval^29^. Trimmed reads were initially mapped to Amel 4.5^17, 26^ using bwa-0.7.5a-r405, with seed length disabled to improve mapping efficiency in ancient DNA datasets^30^. The alignments were sorted using Samtools^31^ and filtered for PCR duplicates using Picard MarkDuplicates-1.88 (https://picard.sourceforge.net), and for paralogs using BWA. We used ANGSD (Analysis of Next Generation Sequencing Data)^32^ for quality filtering and data processing. In all ANGSD analyses, we required a minimum mapping quality of 30 and minimum base quality score of 20. We calculated error rates using an outgroup individual and an error free individual. We randomly selected a modern sample from lineage M from Poland (SRR957058) as the error-free individual, and the outgroup individual used was a modern *Apis cerana* (SRR957079). We used mapDamage^33^ to display nucleotide misincorporation patterns and rescale the quality scores in the bam files. After rescaling we recalculated error rates and compared them with the previous estimates. The rescaled sequences were used in subsequent analyses. We calculated genome-wide coverage in the modern individuals and depth of coverage within the capture regions for both modern and historical individuals. Five historical samples were excluded from further processing, as they had an average coverage below 0.5 ×. Genotype likelihoods were estimated based on the aligned reads and associated mapping, and sequencing quality scores for all individuals.

#### Population structure

We used NGSadmix version 32 to test the number of genetically distinguishable populations in our data^34^. As the presumptive number of evolutionary lineages in *Apis mellifera* is five, we ran NGSadmix for K between two and nine. The evolutionary history of the individuals was inferred using Neighbor-Joining^35^. Haploid genotypes from ancient and modern samples were obtained by randomly sampling one read per position of each of the samples with ANGSD. The tree was built using the program RapidNJ^36^. FigTree v.1.4.4 (Rambaut, 2012; https://tree.bio.ed.ac.uk/software/figtree/) was used to visualize the tree. To compare the genetic diversity of our dataset with other published honeybee datasets, we built two haplotype networks using mitochondrial DNA sequences. In addition to the modern and historical samples, we used other *Apis mellifera* mitochondrial sequences downloaded from the NCBI website (Table 1). All mitochondrial sequences were aligned using MAFFT^37^. Cytochrome b (CytB) and the sequence spanning from the beginning of the Cytochrome Oxidase I to the end of Cytochrome Oxidase II (COI-COII) were extracted separately from the alignment, according to the NCBI sequence KM458618.1^38^. Haplotype networks were reconstructed using TempNet^39^. The procedure was repeated using the non-admixed individuals from subsets as defined in Supplementary Table S1.

**Table 1:** GenBank accession numbers for *Apis mellifera* mtDNA sequences retrieved for use in this study.

GenBank accession number	Subspecies	References	Pubmed ID
KM458618	*Apis mellifera intermissa*	Hu et al^38^	25259457
KJ601784	*Apis mellifera scutellata*	Gibson and Hunt^59^	24708125
NC_001566	*Apis mellifera ligustica*	Crozier and Crozier^60^	8417993
KP163643	*Apis mellifera syriaca*	Haddad^61^	25633178
KJ396191	*Apis mellifera mellifera*	Fuller et al.^62^	26159619
KJ396190	*Apis mellifera mellifera*	Fuller et al.^62^	26159619
KJ396189	*Apis mellifera mellifera*	Fuller et al.^62^	26159619
KJ396188	*Apis mellifera mellifera*	Fuller et al.^62^	26159619
KJ396187	*Apis mellifera mellifera*	Fuller et al.^62^	26159619
KJ396186	*Apis mellifera mellifera*	Fuller et al.^62^	26159619
KJ396185	*Apis mellifera mellifera*	Fuller et al.^62^	26159619
KJ396184	*Apis mellifera mellifera*	Fuller et al.^62^	26159619
KJ396183	*Apis mellifera mellifera*	Fuller et al.^62^	26159619
KJ396182	*Apis mellifera mellifera*	Fuller et al.^62^	26159619

#### Genetic variability and neutrality tests

Based on NGSadmix results for K = 5 and geographical location, we grouped individuals according to the most likely lineage they belonged to: lineage A in South Africa; lineage Y in Kingdom of Saudi Arabia and Yemen ; lineage C in Malta , Italy, Croatia, Slovenia, Austria (AT1971), Switzerland, Bulgaria, Germany, and Denmark; lineage M in Austria, The Netherlands, Sweden, England, France, Luxembourg, Portugal, Spain, Scotland, and Poland; and lineage O in Jordan and Lebanon (see Supplementary Table S1, and Fig. 1 for details). As sampling was done ad-hoc and lineages only ascertained a posteriori, our study does not include modern individuals from lineage O, and ancient ones from lineages A and Y. However, lineages C and M are represented with both historical and modern individuals (Supplementary Table S1). We divide each group further, including four unmixed or less mixed individuals, based on geographic location (Supplementary Table S1).

We estimated the population scaled mutation rate θ and the neutrality test statistic Tajima's D according to the method described in Korneliussen et al.^40^. ANGSD was also used to estimate Watterson and Pairwise θ. We used F-statistics to investigate the genetic distance between the populations observed in NGSadmix. Reynold weighted F_ST_^41^ was calculated using ANGSD, for each of the subgroups defined above. We looked for outlier levels of F_ST_ to identify loci that have probably undergone geographically restricted positive selection^42^. We performed 45 pairwise comparisons, focused on modern versus historical samples in lineages C and M: (i) Historic vs Modern C, (ii) Historic vs Modern M North, and (iii) Historic vs Modern M South.

## Results

In this study, we sequenced 46 historical honeybees from 17 different European countries. Depth of coverage in the targeted regions was between 0 × and 59 ×, with an average of 25.75 × (1.43 × genome-wide [0.0 × to 5.02 ×]). Depth of coverage within the targeted regions was on average 20 times higher than in other sites in the genome, ranging from a threefold to a 66-fold increase (Table 2). Five historical honeybees with depth of coverage below 0.5 × in target regions were excluded from further analysis. Depth of coverage was 7 × in the *Apis cerana* specimen used as outgroup, while in the rest of the modern specimens, depth of coverage ranged from 12 to 27 × (Table 2).

**Table 2:** Depth of coverage and number of sites covered of modern and historical individuals.

Contemporary			Historic				
Name	Overall		Name	Overall		In target regions	
	Average depth	Number of sites		Average depth	Number of sites	Average depth	Number of sites
SRR957058	12.46	196,286,045	AT1954	1.73	195,077,126	27.95	4,567,548
SRR957059	21.97	196,284,708	AT1971	2.79	194,157,924	54.16	2,093,856
SRR957060	23.98	196,284,676	BG1961a	0.74	195,078,785	24.30	4,569,991
SRR957061	20.75	196,285,722	CH1984	0.50	195,082,638	21.13	4,574,740
SRR957062	17.74	196,285,798	CH1986a	0.00	195,083,114	0.00	4,575,698
SRR957063	24.79	196,285,515	CH1986b	1.23	195,080,899	8.68	4,575,531
SRR957064	22.57	196,285,709	DK2013	3.22	194,642,244	59.07	3,324,508
SRR957065	23.70	196,285,142	EN1935	0.80	195,038,026	40.16	4,446,415
SRR957067	22.01	196,285,010	EN1946	0.53	195,082,971	8.26	4,575,698
SRR957069	23.17	196,285,146	EN1961b	1.05	195,070,124	36.71	4,561,347
SRR957070	22.11	196,284,951	ES1960a	0.22	195,082,948	0.64	4,575,698
SRR957071	22.97	196,285,245	ES1973b	0.58	195,080,262	8.80	4,575,698
SRR957072	21.86	196,285,108	ES1973c	1.17	195,076,027	39.91	4,572,024
SRR957073	20.99	196,285,526	ES1973d	1.70	194,725,209	51.33	3,575,185
SRR957074	18.79	196,285,669	ES1973e	1.03	195,075,075	27.75	4,559,578
SRR957075	25.14	196,284,952	FR1955	1.45	195,035,804	37.01	4,452,523
SRR957077	22.70	196,285,502	FR1984b	3.19	194,879,649	56.52	4,015,462
SRR957078	23.94	196,285,407	HR1974	0.04	195,083,113	0.35	4,575,698
SRR957079	7.69	196,285,686	IT1878a	2.58	195,079,797	21.45	4,574,060
SRR957080	27.01	196,284,238	IT1964	1.79	195,081,772	10.07	4,575,656
SRR957081	25.88	196,284,988	IT1976a	1.37	195,049,253	43.18	4,481,224
SRR957082	25.15	196,284,652	IT1976b	0.00	195,083,114	0.06	4,575,698
SRR957083	26.55	196,284,303	JO1978b	5.02	195,070,310	19.33	4,575,441
SRR957084	26.02	196,284,522	JO1978c	1.40	195,078,707	14.41	4,575,488
SRR957085	24.91	196,284,138	JO1978j	0.00	195,083,114	0.01	4,575,698
SRR957086	26.54	196,284,241	LBxxxxb	1.63	195,082,656	6.03	4,575,698
SRR957087	25.08	196,284,123	LU1937a	1.02	195,082,851	18.89	4,575,451
SRR957089	26.00	196,285,648	MT1980a	2.48	194,947,345	52.65	4,192,756
SRR957090	26.43	196,285,686	MT1980c	2.61	194,312,347	55.33	2,469,908
SRR957091	25.66	196,285,203	MT1980d	1.97	194,688,298	50.44	3,488,912
SRR957092	25.64	196,285,460	MT1981	0.02	195,083,114	1.06	4,575,698
SRR957093	24.09	196,285,317	NL1957	0.83	195,082,558	21.20	4,574,888
SRR957094	15.45	196,285,588	NL1968	1.39	195,008,735	45.18	4,366,422
SRR957095	25.29	196,285,464	NL1993b	0.00	195,083,114	0.03	4,575,698
SRR957096	25.94	196,285,343	NL1998	2.55	194,700,060	54.19	3,493,636
			NL1999	2.48	194,271,446	52.41	2,381,308
			PT1967b	1.77	194,910,635	45.85	4,086,547
			PT1984	0.13	195,082,960	6.08	4,575,698
			PT1985	1.98	195,074,317	7.39	4,575,698
			SC1917a	0.86	195,082,776	14.31	4,575,469
			SC1917b	2.20	195,082,865	17.15	4,575,698
			SC1917c	1.08	195,071,369	30.09	4,566,997
			SE1961a	0.80	195,080,667	13.21	4,574,913
			SE1961b	2.40	195,050,275	33.56	4,538,797
			SE1961c	2.58	195,059,343	27.87	4,563,179
			SE1961d	1.05	195,071,084	20.45	4,573,908

### Quality control

Sequences from modern specimens showed relatively low error rates between 0.01% and 0.16%. Historical samples presented higher error rates (between 0.1 and 0.7%), which correspond mainly to post-mortem deamination C->T and G->A (Supplementary Figure S1). These affected mostly the beginning and end of reads, which is to be expected in museum preserved samples. After masking transitions with mapDamage, error rates were halved.

### Phylogeny

**Figure 2:**
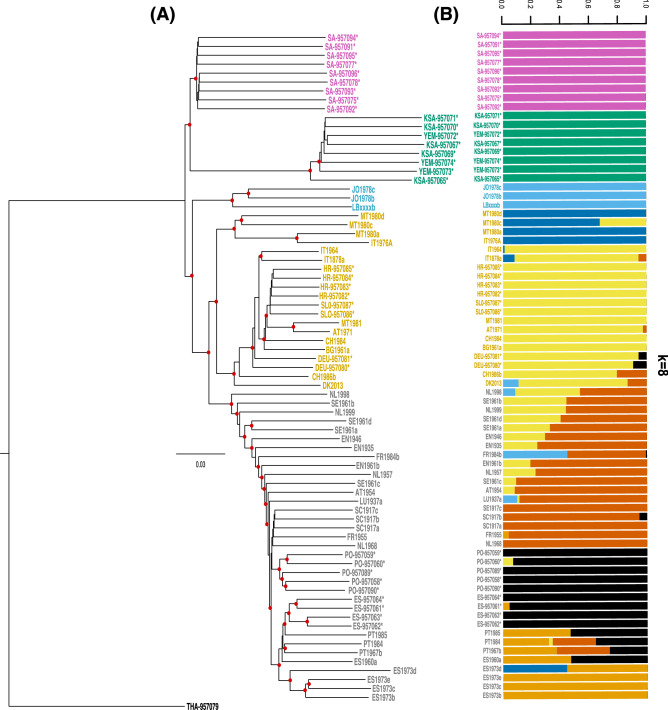
Phylogenetic tree and admixture plot of honeybees (**A**) Neighbor-Joining tree of historic and contemporary (denoted by an asterisk) European honeybees. *Apis cerana* is used as an outgroup. Colour of labels correspond to the five evolutionary lineages: pink—lineage A, green—lineage Y, violet—lineage O, orange—lineage C, and gray—lineage M (**B**) Admixture plot for K = 8. Colour of labels as in (**A**). Coloured bars represent proportion of membership to each K group. When possible, colours match colours in (**A**), such as in lineages A, Y, and O.

Phylogenetic relationships between all the individuals is represented in Fig. 2A, in parallel with the admixture plot for K = 8 (see next section). This value of K was chosen as it mirrors the clades inferred from the phylogenetic tree. There are two main branches: the top one includes the two African lineages: A and Y. The next lineage to split is lineage O, followed by C and M. Finally, lineage C further subdivides in two groups.

**Figure 3:**
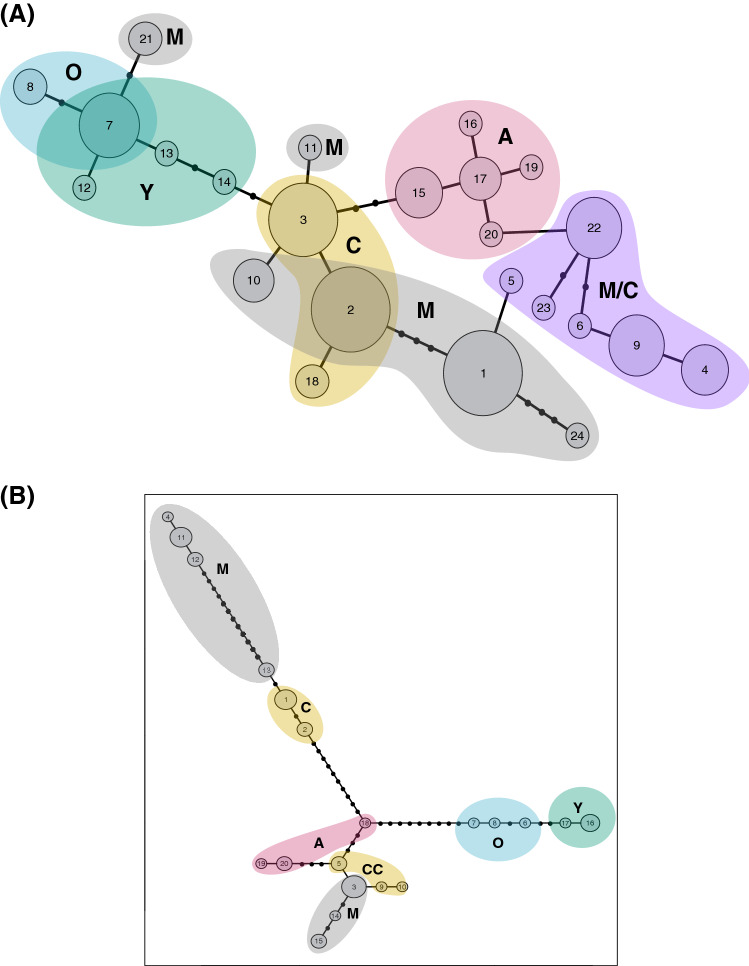
Haplotype network based on (**a**) cytochrome b (CYTB) and (**b**) cytochrome c oxidase 1 and 2 (COI-COII). Size of circles is proportional to number of individuals with that haplotype; black circles represent haplotypes not present in our sampling. Coloured areas represent the evolutionary lineages where enclosed haplotypes have been found.

The haplotype networks obtained from cytochrome b and COI-COII region can be seen in Fig. 3. Both networks show some degree of clustering according to lineages, with individuals belonging to lineages C and M sharing some haplotypes.

### Admixture was widespread

Admixture proportions of both modern and historical samples are represented in Fig. 2B (admixture proportions for other values of K are shown in Supplementary Figure S2). Admixture was high for many historical European samples. Individuals from Scandinavia, and some from the Netherlands, England, and France have high levels of admixture. The level of admixture observed for modern samples was low as previously reported in Harpur et al.^28^, but this does not mean admixture does not occur, as individuals were chosen for that study because they were not admixed.

### Nucleotide diversity was higher in historic populations

**Figure 4:**
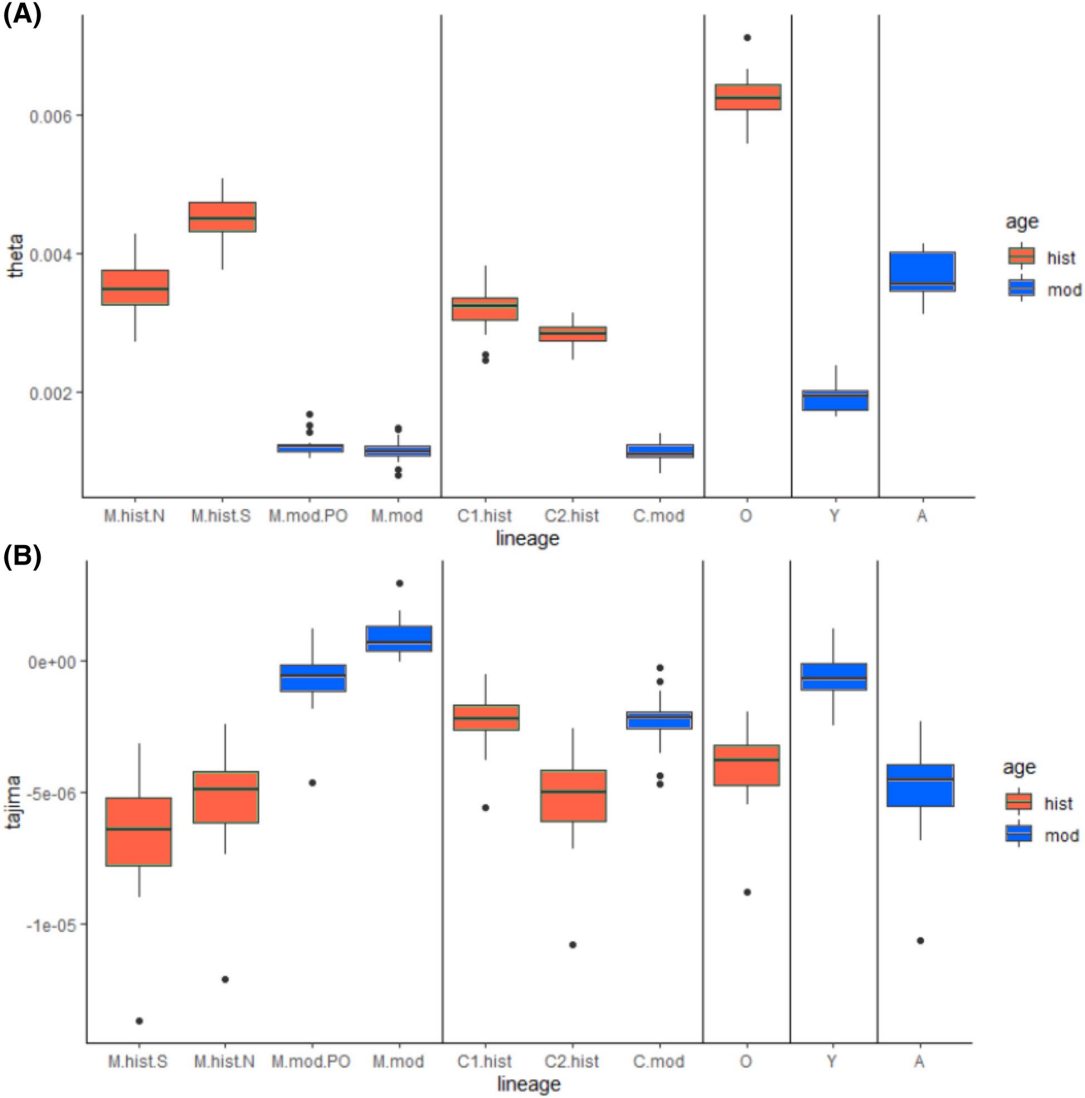
Boxplot of (**A**) Watterson’s estimator and (**B**) Tajima’s D values per chromosome for several groups of European honeybees. Colour indicates historic (red) or contemporary (blue) populations. Alphanumeric codes represent groups of 4 (3 in lineage O) least admixed individuals from a combination geographical area, lineage and time, as described in supplementary Table S1.

Supplementary Figure S3 shows the distribution of Watterson theta across the different chromosomes, and a plot with the values of Tajima's D can be seen in Supplementary Figure S4. Nucleotide diversity is highest in historical O lineage individuals and lowest in the Y lineage samples. Individuals belonging to lineages A and M seem to have similar levels of nucleotide diversity. On the other hand, lineage C has lower levels of diversity but not as low as lineage Y. Boxplots with theta Watterson (Fig. 4A) and Tajima’s D (Fig. 4B) were made using the sites from the haploid sampling and with the group division explained above. The highest level of diversity can be seen in the historical samples from the O lineage. The modern A group has an intermediate level of diversity, whereas the groups formed by historic C and M are close to this A group. Groups formed by modern C and M have low diversity levels. The modern Y group has a slightly higher diversity than the latter.

### Fixation indices follow lineage distributions

Pairwise F_ST_ values are shown in Table 3. The highest values are in the pairwise comparisons of lineage Y with the other lineages. Moderately high are the F_ST_ values for the pairwise comparisons with the modern C group. The distance between the C groups and the M groups is also high despite geographic proximity. Lineage A is more like the historical populations of the C and M groups and to the O group, according to F_ST_ values. Modern samples are more distant to lineage A. The historical C samples are also close to the O group, while the historical M samples from the North are close to all other M samples modern and historical. Furthermore, historical M samples from the North of Europe are most like the modern M samples from Poland, which makes sense geographically. And, similarly, historical M individuals from the South of Europe are like modern M samples from Spain. On the other hand, modern M specimens from Spain and Poland are also close to each other, according to F_ST_ values.

**Table 3:** Pairwise F_ST_ values between subgroups of *Apis mellifera* as defined in the text.

	A	Y	C1-hist	C2-hist	C-mod	M-hist-N	M-hist-S	M-mod	O	M-mod-PO
A										
Y	0.326									
C1-hist	0.214	0.397								
C2-hist	0.284	0.462	0.225							
C-mod	0.350	0.559	0.294	0.097						
M-hist-N	0.279	0.451	0.299	0.349	0.441					
M-hist-S	0.297	0.450	0.318	0.369	0.451	0.143				
M-mod	0.343	0.558	0.387	0.460	0.589	0.155	0.180			
O	0.188	0.327	0.222	0.248	0.307	0.284	0.296	0.355		
M-mod-PO	0.334	0.551	0.373	0.435	0.564	0.109	0.201	0.179	0.344	
Average	0.29	0.45	0.3	0.33	0.41	0.28	0.3	0.36	0.29	0.34

### Genes under selection

We also looked at selection over time. Histograms of number of gene regions per F_ST_ value are shown in Supplementary Figure S5. The top 5% genes with higher F_ST_ values for three pairwise comparisons (historical vs modern C, historical vs modern M east, and historical vs modern M west) were selected (Supplementary Table S3). Two genes are common in the three comparisons: MRJP7, major royal jelly protein, and an unnamed gene that synthesizes a membrane protein (Table 4).

**Table 4:** Genes with the highest 5% Fst values in all pairwise comparisons of historical vs modern *Apis mellifera* populations.

Gene	NCBI ID	Start-Stop	Transcript ID	Function
*MRJP7*	NC_007080.3	2,610,927–2,614,033	GB55213-RA	Part of the Major royal jelly protein/protein yellow family^55^
*Not available*	NC_007070.3	28,394,186–28,396,504	GB42320-RA	Integral component of membrane (UniProt)

See text for details.

## Discussion

### Historic data supports five genomic lineages in honeybees

The topology of the *Apis mellifera* phylogenetic tree which includes both modern and historical samples corroborates previous phylogenetic inferences and its division in five lineages^28,43,44^. In this analysis, we did not compare our data with the sequences from Wallberg et al.^43^, as they used SOLiD sequencing chemistry, and combining data from two different sequencing technologies might have caused biases in our estimates. Nevertheless, Cridland et al.^44^ successfully generated a phylogenetic tree which included representatives of A, C, M, O, and Y lineages based on whole genomes that, globally, is congruent with the phylogeny generated in this study based on targeted regions (Fig. 2A). In both phylogenomic reconstructions, lineage Y seems highly divergent, originating from lineage A shortly after the separation of A/Y from C/M/O. Wallberg et al.^43^ found evidence of admixture in Jordanian O samples, originating from A populations. However, when including Y-lineage samples, Cridland et al. found that this admixture was from lineage Y rather than A^44^. Our specimens from Jordan and Lebanon do not show any sign of admixture when K = 5 or more and are basal in the Neighbor-Joining tree in regard to the European lineages. This would imply that admixture from lineage A is more recent than 1976, when our Jordanian samples were collected, or reflect different sampling localities. Harpur et al.^28^ did not find any significant admixture between lineages in their data; however, this was to be expected giving their sampling strategy to avoid admixed individuals.

### Decrease in genetic diversity in modern honeybees compared to historic populations

Despite the limited sample size, we think that our estimates of genetic diversity are reliable, as both simulation and empirical studies indicate that a large sample size is not required when analysing a large number of SNP markers^45,46^. Our results suggest that genetic diversity has decreased in European honeybees over the last century. This is supported by the lower nucleotide diversity found in modern C-M samples (π ≅ 0.001), compared to historical ones (π ≅ 0.003). Genetic diversity influences a wide range of phenotypes in honeybee colonies, from expression of antimicrobial compounds, resistance to pathogens, thermoregulation, foraging behaviour and colony defence^8^, all essential to colony survival, and response to environmental stress, with lower genetic diversity reducing the variation of these phenotypes as well. Tasks within a colony, such as defence and hygienic behaviour, are performed by a small subset of workers descendent from only some patrilineal lines^8^. Differences in propensity for certain tasks are believed to be influenced by genetics. For example, hygienic and non-hygienic colonies have a difference in gene expression in Cytochrome P450 gene and a limited number of other genes^47^. This means that when genetic diversity is decreased the number of workers in a colony performing some tasks may decrease or less specialized workers will perform such tasks, decreasing the efficiency of the colony^48^. This may originate from high selection pressure selecting for traits based only on queen performance but ignoring the genetic contribution of drones or failing to maintain sufficient levels of genetic diversity within a colony.

We hypothesize that management practices that increase relatedness between colonies, as well as a reduction of number and density of colonies due to, for example, a decrease of suitable habitat availability, are the main factors contributing to the observed decrease of genetic diversity. At the population level, genetic diversity can be affected by selective sweeps, background selection, temporal fluctuations in the direction of selection on segregating alleles^49^, and the level of genetic recombination^48^.

In addition, decreasing density or fewer colonies, demographic expansions, as well as habitat fragmentation would also lower genetic diversity. Colony density in wild populations in Europe is much lower than in African savannahs, despite harsher environmental conditions. This has been associated with more intensive beekeeping in Europe^50^. It has also been found that abiotic factors, such as temperature and land use, are associated with both density of colonies and genetic diversity^51^.

Domestication and professional breeding aim at selecting individuals with specific traits, consciously or unconsciously narrowing genetic variation. Artificial selection on managed hives, however, would only have an indirect effect on wild colonies when drones from managed hives breed with wild queens, or new queens from a managed hive establish a new colony in the wild. This is very frequent, as beekeepers do not track all bees in their colonies. In any case, in many European countries, there are much less wild colonies than managed ones due to the lack of suitable nesting places, so gene flow between them has probably masked any genetic difference between them.

Our results seem to contradict those of a study from 2012^52^ where Harpur and colleagues find the within-colony diversity to be higher in managed colonies than in wild ones, while our results show global patterns of decline within lineages. It should be emphasized that the two are studying different levels of genetic diversity, theirs within-colony diversity in admixed populations, and ours on a meta-population level looking only at non-admixed individuals. It is not surprising that Harpur et al. found higher diversity within managed colonies, as beekeepers will bring in stock from other parts of the world, what they called the progenitor populations, and these will admix with the local populations despite beekeeper’s intentions, creating higher diversity descendants as de la Rua highlights in a reply to that work^53^. Our results indicate that despite this within colony gain, global patterns of diversity decreased within a short time span (from 1960–1984 to 2013 in lineage C and 1917–1973 to 2013 in lineage M).

### Signs of positive selection within the MRJP gene family

We detected signs of selection in MRJP7 and in another gene of unknown function. In a recent genomic analysis, Harpur et al. also detected positive selection in MRJP 7 and MRJP 4^28^, another gene of the same family, in current honeybees. Major Royal Jelly Protein is a family of nine genes and one pseudogene located in tandem on a 60 kb cluster located on chromosome 11^54^. This family of genes encodes secretory proteins that are the major protein content of Royal Jelly, a nutrient-rich substance produced by nurse bees used to feed the larvae, which is only found in some genus of Hymenoptera^55^. MRJP seems to have evolved recently deriving from the *Yellow* family of genes and it seems to have diversified independently in each species where it has been found. In honeybees, MRJP is mostly expressed in workers (particularly nurses) but also other castes^56,57^, and besides being involved in the production of Royal Jelly, it has also been associated with brain function^58^, caste determination and many aspects of eusociality^54^. These functions seem to derive from Royal Jelly’s function in establishing division of labour in the colonies through determining the development of larvae into queens and worker. However, their biochemical function is not determined at the moment. MRJP 4 is down regulated in honeybee heads after infection^54^. Given that Royal Jelly Proteins affect many aspects of behaviour, nutrition and development, and that this pattern of selection is found not only when analysing modern bees alone^28^, but also when comparing historical bees to modern bees, we speculate that domestication can be responsible for the selection signal. While MRJP7 is but one gene within the MRJP/Yellow family, we speculate that selection on this gene, due to its association with nutrition and development, could be caused by selective pressure from beekeeping practices such as the desire for higher honey production or more fertile queens.

The observed decrease in genetic diversity could potentially have an impact on the ability of colonies to react and survive to current and upcoming threats, such as pathogens, pesticides, and climate change. The distribution of evolutionary lineages and admixture proportions in honeybees is not fully understood yet. Several geographical regions are under-sampled, such as most of Africa and areas of the Middle East. The fragmented nature of the sampling carried out in most honeybee genetic studies, has made the lineage nomenclature inconsistent, making comparisons among studies difficult, unreliable or impossible. Mapping with better precision the distribution of each lineage and areas of current and past admixture would help us to better understand the population dynamics of honeybees. Natural history collections with proper annotations of sampling locality and date prove once again to be an essential resource to study temporal trends and provide a glimpse of evolutionary processes occurring in historical times.

## Supplementary information

Supplementary information

Supplementary Table S1

Supplementary Table S2

Supplementary Table S3

## Data Availability

The BAM files of the sequence data mapped to the Apis mellifera genome v4.5 have been deposited in the Short Read Archive under BioProject PRJNA505606. Custom scripts used to analyse the data can be accessed in https://github.com/LuciaRT/code-bees.
