# Supplementary material for: Declining genetic diversity of European honeybees along the twentieth century
Source: Sci Rep. 2020 Jun 29;10:10520. doi: 10.1038/s41598-020-67370-2 (PMC7324561; doi:10.1038/s41598-020-67370-2)
Supplement: Supplementary file 1 — Supplementary information [file 41598_2020_67370_MOESM1_ESM.docx]

**Declining genetic diversity of European honeybees along the 20th century**

Short title: Declining genetic diversity of European honeybees

Gonçalo Espregueira Themudo^a,b,c,1^, Alba Rey-Iglesia^a,d,2,3^, Lucía Robles Tascón^e,2, 4^, Annette Bruun Jensen^f,5^, Rute R. da Fonseca^e,g,6^, Paula F. Campos^a,d,7^

^a^ CIIMAR, Interdisciplinary Centre of Marine and Environmental Research, University of Porto, Avenida General Norton de Matos, S/N, 4450-208 Matosinhos | Portugal

^b^ Section of Forensic Genetics, Department of Forensic Medicine, Faculty of Health Sciences, University of Copenhagen, Frederik V’s vej 11, 2100 Copenhagen, Denmark.

^c^Institute of Biological Psychiatry, Mental Health Centre Sankt Hans, Capital Region of Denmark, 4000 Roskilde, Denmark.

^d^Centre for GeoGenetics, Natural History Museum of Denmark, University of Copenhagen, Copenhagen, Denmark

^e^The Bioinformatics Centre, Department of Biology, University of Copenhagen, Copenhagen, Denmark

^f^Department of Plant and Environmental Sciences, University of Copenhagen, Thorvaldsensvej 40, 1871 Frederiksberg C, Denmark

^g^Center for Macroecology, Evolution and Climate, Natural History Museum of Denmark, University of Copenhagen, Copenhagen, Denmark

# Supplementary Material and Methods

## Subject details

### Collection of historical specimens

Pin-dried specimens of *Apis mellifera* were obtained from museums in Copenhagen (Statens Naturhistoriske Museum, University of Copenhagen) and Aarhus (Naturhistorisk Museum), in Denmark; Leiden (Naturalis Biodiversity Center), The Netherlands; Coimbra (Museu da Ciência, University of Coimbra) and Lisbon (Museu Nacional de História Natural e da Ciência), Portugal. The time span of the specimens is from 1850 to 2002 (See Figure 1 and Supplementary Table S1). An effort was made to cover most of the natural range of honeybees in Europe and include localities within the range of most of the known subspecies, including *A. m. carnica, A. m. ligustica, A. m. iberica, A. m. caucasica, A. m. mellifera, A. m. cecropia, A. m. cypria*, and *A. m ruttneri. Apis mellifera sicula* only occurs on a province in Sicily and it is not clear if the specimen we have from this Mediterranean island belongs to that subspecies. Our sampling covers lineages C, M, and O.

### Post-2006 sequences

## Whole genome sequences of 40 honeybees from lineages A, C, M, and Y and one *Apis cerana* were retrieved from NCBI’s short read archive [23].

## Method details

### DNA extraction

All DNA extractions, library preparations and PCR set-ups were performed in a dedicated ancient DNA laboratory to avoid contamination with environmental DNA, given that endogenous DNA yield was likely to be very low. All subsequent molecular biology laboratory work, such as PCR amplification, Bioanalyzer runs, target capture and sequencing, was performed in a separate DNA facility. Total genomic DNA was extracted using the non-destructive method described in Gilbert et al [17] and detailed in Campos & Gilbert [18]. In this protocol, a whole specimen is submerged in a digestion buffer for 12-24 hours, after which it is removed and placed in 100% ethanol for 2-4 hours, and finally dried and placed back in the collection. DNA was then purified from the digestion buffer using the Minelute, PCR purification kit (Qiagen) following manufacturer’s protocol with the following modification. In elution step, spin columns were incubated in 50µl of EB at 37°C for 10 minutes, spun down and repeated once more to increase DNA yields. The eluates from both rounds of elution were pooled.

### Capture design

We designed the target-capture probes using the latest assembly of the honeybee genome version Amel4.5 (20, 21). To map the distribution of single nucleotide polymorphisms in the different evolutionary lineages we mapped the raw sequence reads of 40 contemporary honeybee genomes [23]. We randomly selected gene locations using coordinates in gene set AmelOGSv3.2 [24], but also ensured that specific genes from the immune [26], sensory [27], and behaviour [28] biochemical pathways were included. A customized probe array consisting of 6,014 individual probes (see Supplementary Table 2) and targeting around 0.7% (~2 million base pairs) of the Honeybee genome was designed and ordered using the MYbaits system (MYcroarray Inc.).

### Library building and sequencing

Blunt-end libraries were built on 21.25 μL of each DNA extract using NEBNext DNA Sample Prep Master Mix Set 2 (New England Biolabs, E6070), following manufacturer’s protocol with modifications as described in Raghavan et al. [29]. Reaction volumes were reduced by a quarter in the end-repair step and by half in the ligation and fill-in steps. Prior to capture, libraries were PCR indexed with six nucleotides barcodes, as described in Meyer & Kircher [30]. The purified libraries were amplified in a two-step manner, where 5 μL PCR product from the first amplification round was transferred into new 50 μL PCR reactions. To increase library complexity, the second-round PCRs were set up with four reactions in parallel. PCR products were then pooled and purified through a single Qiagen MinElute spin column, and eluted in 25-μL EB buffer following 10-min incubation at 37 °C. The purified libraries were amplified with the following conditions: 25 μL DNA library, 1× high-fidelity PCR buffer, 2 mM MgSO4, 200 μM dNTPs each (Invitrogen, Carlsbad, CA, USA), 200 nM Illumina Multiplexing PCR primer in PE 1.0 10 μM, 4 nM Illumina long index primer 10 μM, 1 U of Platinum Taq DNA Polymerase (High Fidelity) (Invitrogen, Carlsbad, CA, USA), and water to 50 μL. Cycling conditions were: initial denaturing at 94 °C for 4 min, 8 cycles of: 94 °C for 30 s, 60 °C for 30 s, 68 °C for 40 s and a final extension at 72 °C for 7 min. A second round of PCR (four parallel reactions for each library) was set up as follows: 5 μL of purified product from first PCR round, 1× high-fidelity PCR buffer, 2 mM MgSO4, 200 M dNTPs each, 200 nM each of Sol_bridge_P5 and Sol_ bridge_P7 10 μM (Maricic, Whitten & Pääbo, 2010), 1 U of Platinum Taq DNA Polymerase (high fidelity) and water to 50 μL. Cycling conditions included an initial denaturing at 94 °C for 4 min, 10 cycles of: 94 °C for 30 s, 58 °C for 30 s, 68 °C for 40 s and a final extension at 72 °C for 7 min.

Libraries were then amplified through PCR so that there was enough material for performing the capture experiment (100-500 ng of each library). For some of the samples, this amplification did not yield enough material, so we performed a second PCR (re-amplification step) to ensure we had the required DNA amounts. The DNA concentration of the amplified libraries was measured in a Qubit 2.0 Fluorometer (Invitrogen, inc.), after having performed a purification step using Minelute spin columns (Quiagen).

Capture experiments were performed as indicated by the manufacturer (MYcroarray inc.), with no modifications to the protocol (version 2.3.1). Captured libraries were amplified and then quantified using a 2100 Bioanalyzer (Agilent) High-Sensitivity DNA Assay. Finally, libraries were pooled with other indexed DNA libraries and sequenced on Illumina HiSeq platforms at the Danish National High-Throughput DNA Sequencing Centre.

##

## Quantification and statistical analysis

### Mapping

Base calling was performed using the Illumina software CASAVA 1.8.2, with the requirement of a 100% match to the 6-nucleotide index used during library preparation. Adapter sequences were trimmed and filtered for N’s and reads shorter than 30 bp were removed using AdapterRemoval [31]. Trimmed reads were initially mapped to *Amel 4.5* using bwa-0.7.5a-r405, with seed length disabled to improve mapping efficiency in ancient DNA datasets [-l 1000; ,32]. The alignments were sorted using Samtools [33] and filtered for PCR duplicates using Picard MarkDuplicates-1.88 ([http://picard.sourceforge.net)](about:blank), and for paralogs using the X1 tag (not equal to 0) as defined by BWA.

### Filtering and data management

We used ANGSD [Analysis of Next Generation Sequencing Data 34] for quality filtering and data processing, as it accepts as input mapped reads and imputed genotype probabilities. Most of the methods within ANGSD rely on genotype uncertainty, which is very useful for low and medium depth data, generally the case in historic degraded material. In all ANGSD analyses, we required a minimum mapping quality of 30 and minimum base quality score of 20.

### Error rates

We calculated the error rate of the BAM files in ANGSD using an outgroup individual and an error free individual. This error estimation assumes that each sample and the error-free individual should have the same expected number of derived (mutant) alleles, while the extra observed derived alleles in the sample are due to an excess of sequencing errors. Only positions where there is coverage for the outgroup individual, the sample, and the error free individual are used. We randomly selected a modern sample from lineage M from Poland (SRR957058) as the error-free individual, and the outgroup individual used was a modern *Apis cerana* (SRR957079). Consensus FASTA files using the most common base (options -doCounts 1 -doFasta 2) were made for the error-free and the outgroup individuals. ANGSD uses genome information in the BAM header to determine the length and chromosome names. For sites without data an "N" is written. In the case of a tie a random base is chosen among the bases with the same maximum counts. N's or filtered bases are ignored.

### Damage

Ancient DNA samples will typically exhibit a high rate of transition substitutions due to post-mortem deamination; therefore, mapDamage [35] was used to display nucleotide misincorporation patterns. We also used madDamage to rescale the quality scores in the bam files. In the rescaled bam files, quality scores for the potentially damaged sites are lowered so that those sites are not used in further analyses. After this rescaling we calculated a new error rate and compared them with the previous estimates. The rescaled files were used in subsequent analyses.

### Depth of coverage

We calculated genome-wide coverage in the modern individuals and depth of coverage within the capture regions for both modern and historical individuals. Overall coverage was calculated in ANGSD with the options: -GL 1 -minQ 20 -minMapQ 30 -doCounts 1 -maxDepth 200 -nThreads 8 -doDepth 1. To calculate the coverage within the capture regions we used the same options as before, with the addition of the filter -rf specifying the coordinates for the capture regions. After that, average (overall and capture regions) coverage per individual was extracted using an in-house python script. An R script was used to plot the average coverage in the whole genome versus the coverage in the capture regions. Five historical samples were excluded from further processing, as they had an average coverage below 0.5.

### Population Structure

Genotype likelihoods were estimated based on the aligned reads and associated mapping and sequencing quality scores for all individuals (ANGSD options: -GL 1 -doGlf 2 -minQ 20 -minMapQ 30 -minInd 70). We used NGSadmix version 32 to test the number of genetically distinguishable populations in our data. [36]. As the presumptive number of evolutionary lineages in *Apis mellifera* is five, we ran NGSadmix for K between 2 and 9. The software does not allow making cross-validation to determine the number of populations, K [37]. For each successive value of K, the analysis ran up to 2000 iterations. We considered that each analysis converged if the top 10 highest likelihood for a K was within 2 likelihood units.

### Phylogenetic tree

The evolutionary history of the individuals was inferred using Neighbor-Joining [38]. Haploid genotypes from ancient and modern samples were obtained by sampling one read per position of each of the samples with ANGSD. If multiple sequence reads overlapped a position, one read was randomly sampled. This avoids biasing for or against heterozygotes and renders all the samples haploid. We kept only diallelic positions and set minor allele count equal to 2. The resulting file was converted to FASTA format. The tree was built using the program RapidNJ [39] with the options -b 1000 -c 10. FigTree (Rambaut, 2012; http://tree.bio.ed.ac.uk/software/figtree/) was used to visualize the tree.

### Haplotype network

To compare the genetic diversity of our dataset with other published honeybee datasets, we built two haplotype networks using mitochondrial DNA sequences. First, we extracted the mitochondrial DNA in FASTA format using ANGSD with the option -doFasta 2 and specifying the region corresponding to the mitochondria. In addition to the modern and historical samples, we used other *Apis mellifera* mitochondrial sequences downloaded from the NCBI website (Table 1). All of the mitochondrial sequences were aligned using MAFFT [40]. Cytochrome b (CytB) and the sequence spanning from the beginning of the Cytochrome Oxidase I to the end of Cytochrome Oxidase II (COI-COII) were extracted separately from the alignment, according to the NCBI sequence KM458618.1 [41]. Only diallelic sites were kept and the minor allele count was set to 5 (both for the CytB and COI-COII regions). Individuals with a proportion of missing information higher than 0.5 were removed. In the resulting alignment, there were 30 CytB and 50 COI-COII segregating sites. Haplotype networks were reconstructed using TempNet [42]. The procedure was repeated using the non-admixed individuals from subsets as defined in Supplementary Table 1.

### Genetic variability and neutrality tests

Based on the NGSadmix results for K=5 and geographical location, we grouped individuals according to the most likely lineage they belonged to: lineage A in South Africa (SA); lineage Y in Kingdom of Saudi Arabia (KSA) and Yemen (YEM); lineage C in Malta (MT), Italy (IT), Croatia (HR), Slovenia (SLO), Austria (AT1971), Switzerland (CH), Bulgaria (BG), Germany (DE), and Denmark (DK); lineage M in Austria (AT), The Netherlands (NL), Sweden (SE), England (EN), France (FR), Luxembourg (LU), Portugal (PT), Spain (ES), Scotland (SC), and Poland (PO); and lineage O in Jordan (JO) and Lebanon (LB).

As sampling was done *ad-hoc* and lineages only ascertained *a posteriori*, our study does not include modern individuals from lineage O, and ancient ones from lineages A and Y. However, lineages C and M are represented with both historical and modern individuals (Supplementary Table 1). To avoid biases due to number of individuals or high levels of admixture, we divided each group further including 4 non-admixed or least admixed individuals, based on geographic location (Supplementary Table 1). The final groups are C1Hist (historical samples from lineage C being closer to the O group according to the admixture results), C2Hist (historical samples from lineage C), C-mod (modern samples from lineage C), M-hist-N (historical samples from lineage M coming from Scotland and France), M-hist-S (historical samples from lineage M coming from Spain), M-mod (modern samples from lineage M from Spain), and M-mod-PO (modern samples from lineage M from Poland). We estimated the population scaled mutation rate *θ* and the neutrality test statistic Tajima's D according to the method described in Korneliussen et al. [43]. This method calculates site specific estimates of *θ* based on an empirical Bayes approach as implemented in ANGSD using the GATK genotype likelihood model. In brief, it first estimates a global site frequency spectrum (SFS) [43,44] and then calculates posterior sample allele frequencies using the global SFS as a prior. ANGSD was also used to estimate Watterson and Pairwise *θ.* Based on these, Tajima's D and various other neutrality test statistics were calculated for each of subgroups. ANGSD outputs *θ* per chromosome, and given that the mutation rate is per site, we calculated theta per site by dividing by the number of informative sites. Furthermore, we calculated the average of each theta across all chromosomes for each population.

### Fixation Index (F_ST_)

We used F-statistics to investigate the genetic distance between the populations observed in NGSadmix. Reynold weighted F_ST_ [45] was calculated using ANGSD, by calculating sample allele frequency for each population, calculating 2d sfs (2-dimensional site frequency spectrum) for each pair of populations using realSFS, and extracting the F_ST_ values. The seven subgroups described in the previous section were used in the F_ST_ analyses. ANGSD implements pairwise F_ST_ tests between two populations at a time, so 45 pairwise combinations were performed.

### Selection

## Positive selection of certain loci is indicated by outlier levels of genetic differentiation (F_ST_) in relation to the rest of the genome [46]. We looked for these outlier levels of F_ST_ to identify loci that have probably undergone geographically restricted positive selection. We performed 45 pairwise comparisons, focused on modern versus historical samples in lineages C and M: (i) C2hist — Cmod, (ii) M-mod-PO — M-hist-N (Northern European lineage M), and (iii) M-mod — M-hist-S (Southwest European lineage M)

# Supplementary Information titles and legends

Supplementary Table S1 – List of sampling localities, with collection year and geographical coordinates, and group assignments as mentioned in the text.

Supplementary Table S2 – List of probes designed for the target capture of Apis mellifera, including group membership in the Amel4.5 genome assembly, and probe start.

Supplementary Table S3 – List of top 5% *Apis mellifera* genes with the highest values of Fst in three comparisons: Historic vs Modern C lineage; Historic vs Modern M lineage North; and Historic vs Modern M lineage South

Supplementary Figure S1 – Deamination patterns of DNA sequences in historic honeybees at the 5’ (left) and 3’ (right) ends. Each line represents one individual.

Supplementary Figure S2 - Admixture proportions for K = 3 - 7; and 9.

Supplementary Figure S3 - Distribution of Watterson theta across chromosomes among different evolutionary lineages of *Apis mellifera.*

**

Supplementary Figure S4 - Values of Tajima's D across chromosomes among different evolutionary lineages of *Apis mellifera*.

Supplementary Figure S5 – Histogram of gene regions per value of F_ST_ for comparison of modern and historic honeybee populations in Europe. A – Historic vs Modern C lineage; B – Historic vs Modern M lineage North; C – Historic vs Modern M lineage South.

Figure S5 - A

Figure S5 - B

Figure S5 – C
